# Resting-state electroencephalogram microstate to evaluate post-stroke rehabilitation and associate with clinical scales

**DOI:** 10.3389/fnins.2022.1032696

**Published:** 2022-11-18

**Authors:** Zhongpeng Wang, Zhaoyang Liu, Long Chen, Shuang Liu, Minpeng Xu, Feng He, Dong Ming

**Affiliations:** ^1^Department of Biomedical Engineering, College of Precision Instruments and Optoelectronics Engineering, Tianjin University, Tianjin, China; ^2^Academy of Medical Engineering and Translational Medicine, Tianjin University, Tianjin, China; ^3^Tianjin International Joint Research Center for Neural Engineering, Tianjin, China

**Keywords:** resting-state EEG, microstate analysis, post-stroke, rehabilitation assessment, clinical scales

## Abstract

**Introduction:**

Stroke is usually accompanied by a range of complications, like post-stroke motor disorders. So far, its evaluation of motor function is developed on clinical scales, such as Fugl-Meyer Assessment (FMA), Instrumental Activities of Daily Living (IADL), etc. These scale results from behavior and kinematic assessment are inevitably influenced by subjective factors, like the experience of patients and doctors, lacking neurological correlations and evidence.

**Methods:**

This paper applied a microstate model based on modified k-means clustering to analyze 64-channel electroencephalogram (EEG) from 12 stroke patients and 12 healthy volunteers, respectively, to explore the feasibility of applying microstate analysis to stroke patients. We aimed at finding some possible differences between stroke and healthy individuals in resting-state EEG microstate features. We further explored the correlations between EEG microstate features and scales within the stroke group.

**Results and discussion:**

By statistical analysis, we obtained significant differences in EEG microstate features between the stroke and healthy groups and significant correlations between microstate features and scales within the stroke group. These results might provide some neurological evidence and correlations in the perspective of EEG microstate analysis for post-stroke rehabilitation and evaluation of motor disorders. Our work suggests that microstate analysis of resting-state EEG is a promising method to assist clinical and assessment applications.

## Introduction

Stroke is usually accompanied by a range of complications, one of the recognized post-stroke complications is motor disorder (Handley et al., 2009). Although dyskinesia occurs uncommonly in adult stroke patients, about 1–4% of patients have dyskinesia after stroke severely affecting their life of patients (Bansil et al., 2012; Mehanna and Jankovic, 2013).

There are two clinical scales for motor function evaluation. The first one is the Fugl-Meyer Assessment (FMA), which assesses sensory-motor disorders in stroke patients. FMA has good consistency, responsiveness, and accuracy and is most widely used in clinical assessment (Gladstone et al., 2002). The other is the Instrumental Activities of Daily Living (IADL) assessment, which assess IADL functions in eight categories: shopping in the street, going out, food preparation, household maintenance, laundry, ability to use the telephone, taking medication, and ability to handle finances (Lawton and Brody, 1969). Both scales are based on behavioral scores, which are inevitably influenced by subjective factors, like the experience of patients and doctors, lacking neurological correlations and evidence.

Electroencephalogram (EEG) is an electrical field recording of the cerebral cortex (Nunez and Srinivasan, 2006), where the large-scale activity of cortical neurons creates a specific distribution of electrical fields in the cortex (Schmidt, 1983)and through volume conduction (Helmholtz, 1853), a potential distribution is created on the scalp surface through the skull and scalp (Michel and Murray, 2012). Placing electrodes on the scalp surface, changes in scalp surface potential distribution can be recorded and used to assess the spatial and temporal dynamics of brain electrophysiological activity (Rappelsberger, 1978). EEG is non-invasive, easy to use, and inexpensive, so it is a quite common tool for studying brain activity. EEG signals have a high temporal resolution (Michel and Brunet, 2019) compared to other neuroimaging tools, such as functional magnetic resonance imaging (fMRI). However, the presence of volume conduction affects the spatial resolution of EEG (Michel and Murray, 2012), and the high spatial dimension of EEG signals cannot be interpreted directly, so it is difficult to extract valuable information from EEG data.

There are many methods of EEG analysis used to extract feature information, and microstates analysis is one of them. A microstate is a sub-stable pattern of cortical potential distribution in space and time (Lehmann et al., 1987). In the temporal dimension of EEG, it can be observed that the temporal sequence of EEG topography consists of a set of discrete prototype topographies, each of which maintains a sub-stable state for approximately 60–120 ms before shifting to another prototype topography (Michel and Koenig, 2018). These prototypical topographies are called functional microstates, and in resting-state EEG, microstate topographies share a high similarity across individuals (Koenig et al., 1999), which ensures the feasibility of consistent microstate analysis among subjects. At present, microstate analysis is widely used, such as in the fields of Alzheimer’s disease, schizophrenia, and depression research (Michel and Koenig, 2018), but it is rarely used for motor disorder analysis in stroke.

We applied this method to explore the feasibility of applying microstate analysis to stroke patients. Our work is to find some possible differences between stroke and healthy individuals in resting-state EEG microstate features, and furthermore, to find some correlations between EEG microstate features and scales within stroke patients. It expects to obtain some significant results which can provide some neurological evidence or correlations in the perspective of EEG microstate analysis for post-stroke rehabilitation and evaluation of motor disorders.

## Materials and methods

### Subjects

Our study included 12 patients (nine males and three females, with an average age of 59.8 ± 12.8) with different degrees of stroke motor disorder and 12 healthy subjects (eight males and four females, with an average age of 29.0 ± 7.1). All subjects gave signed informed consent. This study has been approved by the Ethics Committee of Tianjin University and Tianjin Hospital in Tianjin, China.

### Experimental setup

For both groups, resting EEG was collected with eyes open, and additional FMA scale and IADL scale scores were collected in the patient group with stroke motor disorders. Clinical diagnosis was assessed by experienced doctors. The experiment was performed in a quiet room. Subjects were comfortably seated in a chair, and they were asked to rest for a period to meet the standard of resting state EEG. Subjects were asked to keep still to avoid artifacts such as myoelectricity. The EEG data were acquired while remaining resting in the open-eyed state, and record each subject’s EEG for more than 200 s. For patients, the clinical diagnosis was evaluated by an experienced doctor after data collection, then FMA and IADL scores were given. FMA has three scores: total score, upper limb score, and lower limb score.

### Data acquisition and preprocessing

Eye-opened resting-state EEG data were acquired from a 64-channel electrode cap with 64 scalp electrodes (Ag-AgCl) placed according to the 10–20 electrode system of the International Federation of Clinical Neurophysiology (Klem et al., 1999). A 64-channel SynAmps2 system (NeuroScan Inc., USA) was used for EEG recording. The sampling rate was 1,000 Hz. The impedance for all electrodes was kept below 10 kΩ.

Electroencephalogram data were preprocessed offline on MATLAB (R2021b, MathWorks Inc., USA) with the EEGLAB (Delorme and Makeig, 2004) 2022.0 toolbox.

First, down-sampling was performed, and the sampling rate was reduced to 250 Hz, for decreasing the amount of data and the computational stress of data processing. The sampling rate satisfies Nyquist’s sampling theorem (Landau, 1967), and 250 Hz is much larger than twice the 45 Hz.

Second, a Finite Impulse Response (FIR) filter with a filtering range of 1–45 Hz was applied to eliminate low-frequency signal shifts and high-frequency interference.

Third, an Independent Component Analysis (ICA) was performed. After the ICA decomposition of the components, the joint use of ICLabel (Han et al., 2019) and ADJUST (Mognon et al., 2011) algorithm assists in identifying artifactual components, such as blink, muscle movement, eye movement, and electrode loosening components. After that, use EEGLAB’s clear raw data and Artifact Subspace Reconstruction (ASR) function to clean up bad channels and abnormal data segments, then do ICA on the data again, use ICLabel and ADJUST algorithm to assist in identifying artifact components, and visually remove most of the other artifact components.

After the last ICA processing, most of the components in the top ranking of ICA components are brain-active components, and their confidence level can reach more than 90%.

### Data analysis

**FIGURE 1:**
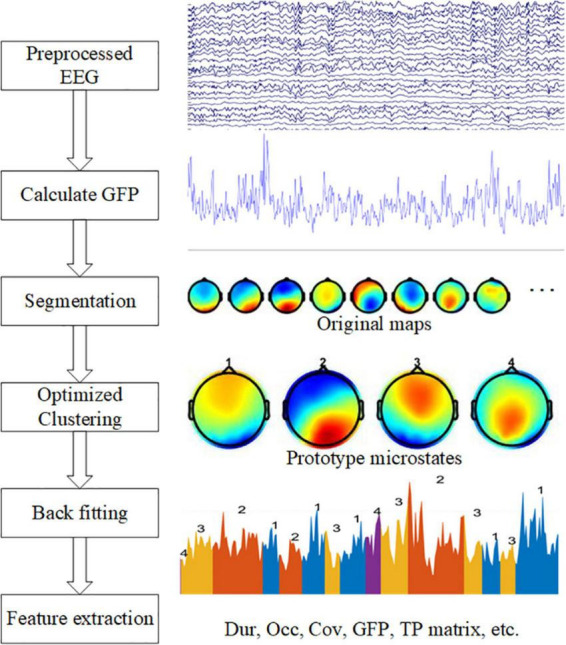
Graphical representation of the analysis method in the study. The left side shows five steps: calculate GFP, EEG segmentation, optimized clustering, back fitting, and feature extraction. On the right side are examples of each step.

We performed microstate analysis to extract features from data using the microstate toolbox (Poulsen et al., 2018), and then performed a statistical analysis of the features. The complete analysis graphical representation shows in Figure 1.

#### Microstate analysis

A common average reference (CAR) is helpful before microstate analysis (Ludwig et al., 2009). Then, the largest signal-to-noise ratio point is determined by calculating the Global Field Power (GFP) of each topography in time series, which is calculated as follows:


$$GFP=\sqrt{\frac{\sum_{i}^{N}{\left(V_{i}(t)-V_{mean}(t)\right)}^{2}}{N}}$$

where _*V_i_(t)*_ denotes the instantaneous potential value of the No. _*i*_ electrode at time _*t*_; _*V_mean_ (t)*_ represents the average potential value of all electrodes at time _*t*_; _*N*_ is the number of electrodes.

Second, the EEG data was segmented. Before segmentation, EEG data should be normalized. The time point where the GFP local maximum was located often indicated the presence of a stable microstate, so the brain topography map at the time point of the GFP local maximum was extracted, these maps were also called original maps. The extracted brain topographies were clustered and analyzed using a modified K-means clustering algorithm (Pascual-Marqui et al., 1995), and the prototype microstates formed by clustering were ranked based on the global explained variance. The clustering operation was repeated using the algorithm for clustering parameters 3–8, respectively.

Third, Optimization of clustering parameters. Usually, the optimization of the clustering parameters is based on the cross-validation criterion (CV), Global explained variance (GEV), Dispersion (W), and Krzanowski-Lai (KL) criterion to choose the suitable number of prototype microstates (Poulsen et al., 2018). However, according to the previous experience (Koenig et al., 1999), the number of resting-state microstates clustering is generally chosen to be 4, because the topography of four prototype microstates has high similarity among different studies, and the fixed microstate clustering number of 4 can ensure the consistency and comparability between individual studies (Michel and Koenig, 2018). These prototype microstate topographic maps are easily recognized, and therefore they are labeled as four classes A, B, C, and D, which are left-right direction (type A), left-right direction (type B), anterior-posterior direction (type C), and frontocentral maximum (type D).

When we got the four microstates, a back fitting was performed based on the number of microstates selected. After the four prototype microstates are obtained by clustering, the whole data set is fitted to the prototype microstates, and each segment of EEG data is matched with a microstate label, which is one of the four prototype microstates. A winner-take-all method was used, i.e., the prototype microstate with the highest similarity is selected as the microstate label of the EEG data and a sequence of microstates is formed. However, the presence of interference and artifacts in the original data affects the quality of the microstate back fitting and may produce some microstates with short durations, which are not true microstates and do not meet the requirements of sub-stability. Therefore, these short microstates need to be rejected, temporal smoothing was performed by merging data segments with microstate durations of less than 30 ms.

In the last step, the microstate features are calculated. For each microstate, the duration (Dur) is defined as the average duration of the microstate per second. Occurrence (Occ) frequency was defined as the average frequency of the observed microstates. Coverage (Cov) was defined as the percentage of each microstate that occurred in each period. The average GFP is defined as the average amplitude of GFP during each microstate class dominance. The transition probability (TP) is defined as the probability of moving from one microstate to another different microstate.

#### Statistical analysis

Statistical analysis was performed on SPSS software (IBM Inc., USA). In this study, the significance level is 0.05. All statistical tests are two-tailed tests.

We conducted an independent sample *t*-test to find whether there are some differences between the features of resting-state patients’ microstates and those of healthy subjects. Then we conducted a Pearson correlation analysis to analyze the correlation between EEG microstate features and scale scores within the stroke group. Considering that the features of microstates may be affected by factors such as age, further partial correlation analysis was performed with age as the control variable.

## Results

### Descriptive statistics

**FIGURE 2:**
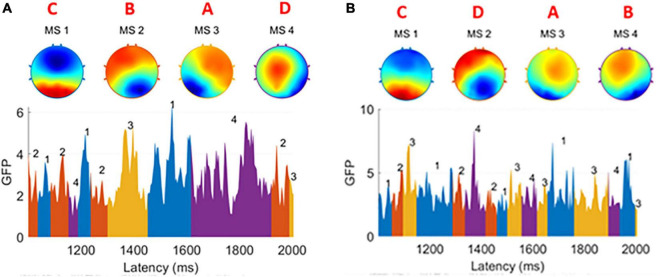
**(A)** Four prototype microstates extracted from one healthy subject. **(B)** Four prototype microstates extracted from one patient subject. The brain topographies are labeled as type A, B, C, and D. In both graphs GFP, the EEG data is divided into small segments. Each of them is corresponding to one of four microstates and a microstate sequence is formed. There are slight differences between the healthy group and the patient group.

In both groups, the number of clusters was set to 4. The cluster maps obtained from each subject’s EEG were highly consistent (Michel and Koenig, 2018), so we called them prototype microstates, yet slight differences could still be found when comparing patients and healthy subjects as Figure 2 shows. We have marked the type of prototype microstates manually. In the EEG time series, the EEG signal is segmented corresponding to each prototype microstate, and each prototype microstate lasts for about 60–120 ms. The topography of the microstates was slightly different between patients and healthy subjects.

**FIGURE 3:**
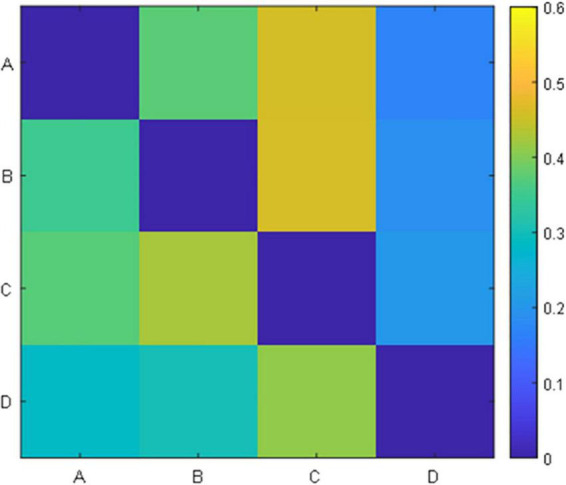
Transition probability matrix.

For each prototype microstate, we calculated the features corresponding to each prototype microstate and grouped them according to the label of the microstate. Each microstate corresponds to four features: average duration, average occurrence, coverage, and average GFP. The features between microstates are the transition probabilities, 12 values in total, they are A-B (A-B denotes the transfer from the prototype microstate A to the prototype microstate B unidirectionally, the same below), A-C, A-D, B-A, B-C, B-D, C-A, C-B, C-D, D-A, D-B, D-C, respectively. A TP matrix was formed with a zero diagonal because the prototype microstate transfer to itself is not meaningful. Table 1 and Figure 3 show the data on the microstate features of one of the patients.

**TABLE 1:** Microstate features GFP, Occ, Dur, and Cov for each prototype microstate.

Microstate features	A	B	C	D
GFP	2.5429	2.6814	2.9412	2.5121
Occ	3.0817	3.3100	3.7209	1.9061
Dur (ms)	79.1481	85.1345	92.6626	67.9401
Cov	0.2439	0.2818	0.3448	0.1295

### Independent samples *t*-test

**FIGURE 4:**
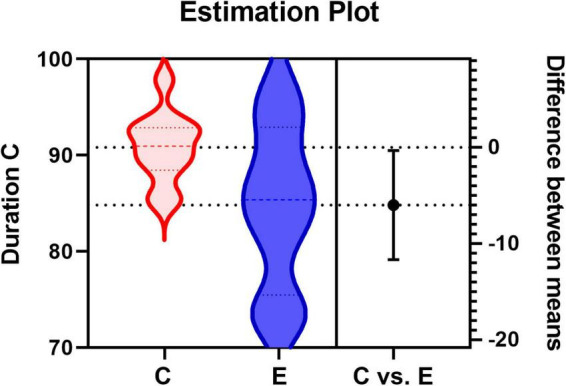
Estimation Plot for the average duration of microstate C. C represents the control group (healthy group) and E represents the experimental group (patient group).

To find differences between the health and patient group, we did independent samples *t*-test. There are two group settings: the experimental group (healthy subjects) and the experimental group (patients), with a total of 28 test variables, i.e., 12 probabilities of transition and a total of 16 (4*4) microstate features corresponding to each prototype microstate. When performing the independent samples *t*-test, it was found that the two groups of data did not meet the requirement of homogeneity of variance, so Welch’s method was used for correction. The following Table 2 was obtained, labeling the differences between the two groups for each microstate feature respectively, and the average duration of microstate C (Dur_C) was significantly different between the groups (_*t=2.268*,  p=0.038_). The detailed information is shown in Figure 4. To examine the influence of age, we applied a two-factor ANOVA analysis to the data, then, we got the result that the factor age has no statistical significance (*F* = 0.782, *p* = 0.387) and the factor Dur_C has a statistical significance (*F* = 5.077, *p* = 0.036).

**TABLE 2:** Independent samples *t*-tests for 28 variables respectively with *t*-values and significance *p*-values.

Characters	A-B	A-C	A-D	B-A	B-C	B-D	C-A
*t*-value	–0.696	1.511	–0.552	–0.227	1.360	–1.009	0.530
Significance	0.499	0.146	0.587	0.823	0.189	0.325	0.602

**Characters**	**C-B**	**C-D**	**D-A**	**D-B**	**D-C**	**GFP_A**	**GFP_B**

*t*-value	0.546	–0.843	–0.390	–0.802	0.978	–0.312	–0.131
Significance	0.591	0.409	0.700	0.433	0.344	0.759	0.897

**Characters**	**GFP_C**	**GFP_D**	**Occ_A**	**Occ_B**	**Occ_C**	**Occ_D**	**Dur_A**

*t*-value	0.042	–0.444	–0.409	–0.414	1.439	–0.859	–0.362
Significance	0.967	0.663	0.687	0.684	0.168	0.401	0.722

**Characters**	**Dur_B**	**Dur_C**	**Dur_D**	**Cov_A**	**Cov_B**	**Cov_C**	**Cov_D**

*t*-value	–0.393	2.268	–0.018	–0.522	–0.564	1.907	–0.716
Significance	0.700	0.038*	0.986	0.607	0.582	0.075	0.482*

GFP_A represents the character GFP of microstate A, and similar definitions as the table show (*represents *p* < 0.05, **represents *p* < 0.01).

### Scale correlations

Pearson correlation analysis was performed using SPSS 25 software to analyze the correlation between the microstate features and the clinical scales. The dependent variables are the scale scores: FMA, upper limb score, lower limb score, and IADL score. The independent variables are the microstate features corresponding to each prototype microstate: average GFP, average duration, average occurrence, coverage, and transition probabilities. Statistical analysis showed a significant correlation between the transition probability from C to D and the scale scores.

**FIGURE 5:**
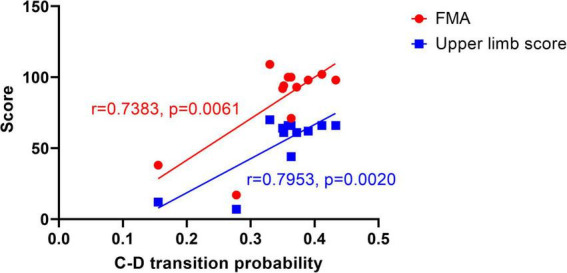
Linear regression graph between C-D transition probability and scores. The dots represent the FMA score, and the squares represent the upper limb score.

There was a significant correlation between C-D transition probability and FMA score within the confidence interval of 95% (*r* = 0.7383, *p* = 0.0061, two-tailed test), which indicates that C-D transition probability influences the FMA score. Furthermore, the C-D transition probability was significantly correlated with the upper limb score (*r* = 0.7953, *p* = 0.0020, two-tailed test) within the confidence interval of 95%. It is noted that the *r*-value of the latter correlation test is higher, and the *p*-value is smaller, suggesting that the C-D transition probability reflects the upper limb score and implies the mobility of the upper limb. Since the microstate features are also influenced by age and gender (Hu and Zhang, 2019), we further implemented a partial correlation analysis, which was performed mainly on the age variable. By setting the control variable as age, there was a significant correlation between C-D and FMA (*r* = 0.742, *p* = 0.009), and C-D and upper limb score (*r* = 0.802, *p* = 0.003) also had a significant correlation. The comparison revealed that age had an insignificant effect on the correlation, which indicates that age is not a major factor affecting the scores and that there is a significant correlation between the transition probability C-D and the FMA score, as well as the upper limb score. For the other microstates, no significant correlation was found. Then we did a linear regression as Figure 5 shows. For transition probability C-D vs. the FMA score, the R squared is 0.5451; for transition probability C-D vs. upper limb score, the R squared is 0.6325.

**FIGURE 6:**
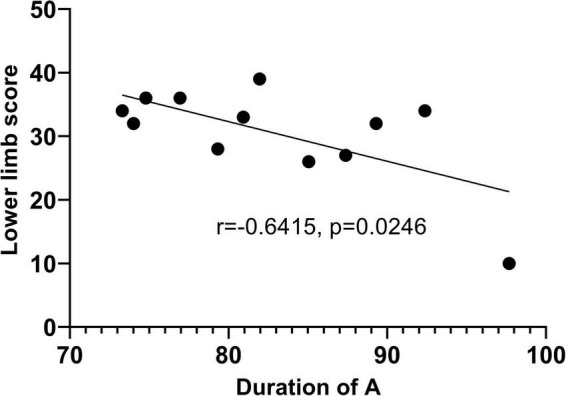
Linear regression graph between duration of microstate A and lower limb score.

The resting state microstate feature average duration of microstate A(Dur_A) was significantly correlated with the lower limb score (*r* = −0.6415, *p* = 0.0246) as Figure 6 shows. Furthermore, partial correlation analysis was performed with the control variable age, and there was statistically significant (*r* = −0.673, *p* = 0.023), suggesting that the correlation between Dur_A and the lower limb score was significant when influenced by the age variable. Linear regression was done, and the R squared is 0.4115.

## Discussion

We used microstate analysis to assess the EEG differences between patients and healthy subjects, and further assessed the correlations between patient microstate features and clinical scales. Our study got statistically significant results. The transition probability from A to C was slightly decreased in patients with stroke motor disorders compared to healthy subjects and the variance was larger within the patient group compared to the healthy group. There was a significant correlation between transition probability from C to D and the FMA score, upper limb score. Particularly, a more significant correlation between transition probability from C to D and upper limb score was found. There was a significant correlation between the average duration of microstate A and the lower limb score. For other microstate features, no significant difference was found between groups, and no correlation was found between other features and other clinical scale scores.

Microstates provide information on the combinatorial activity of large-scale neural networks, and correlations between EEG microstates and fMRI resting states are found in previous studies (Britz et al., 2010; Musso et al., 2010; Yuan et al., 2012). Microstate A correlates with negative Blood Oxygen on Level Depending (BOLD) activation in the bilateral superior temporal lobe and middle temporal lobe, microstate B correlates with negative BOLD activation in the bilateral occipital cortex. Microstate C correlates with positive BOLD activation in the dorsal anterior cingulate cortex, bilateral inferior frontal cortex, and right insula area. Microstate D is associated with negative activation of BOLD in frontal and parietal cortical righting dorsal and ventral areas. Britz et al. (2010) correlate microstate C with activity in cognitive control networks (mainly salience networks) and with activation of the anterior cingulate and insula (Seeley et al., 2007). According to (Britz et al., 2010), microstate D is associated with the focal attention network.

There is an antagonistic relationship between microstate C and microstate D, creating a dynamic equilibrium (Santarnecchi et al., 2017). When a stroke occurs, the equilibrium is disrupted, and the degree of this imbalance varies depending on the severity of the stroke motor disorder. As seen in the figure, the lower the patient score is, the lower the transition probability C-D is, representing a state transition from microstate C to microstate D is less likely to occur. This suggests that the connectivity of the microstate C salience network to the microstate D dorsal attention network is reduced. However, there is no evidence to determine the relationship between changes in network connectivity balance and stroke motor disorder. This may be related to impaired proprioception, which requires a more detailed study of the relationship between microstates and fMRI of patient brain function.

Our study involved a limited number of subjects, more healthy subjects and patients could be involved to get furthermore detailed analysis in the future. However, it suggests the feasibility of applying microstate analysis to stroke patients and that there are some correlations between microstate features and clinical scales. As a purely phenomenological concept, the association between microstates and brain activity remains vague, and more work needs to be done in the future to reveal the association between microstate features and brain activity and to find more neurological evidence.

## Conclusion

In summary, we found some differences between stroke and healthy individuals in resting-state EEG microstate features, which is statistically significant by independent samples *t*-test. And we performed the Pearson correlation analysis between EEG microstate features and scales within the stroke group. We obtained significant differences in EEG microstate features between the stroke and healthy groups, and significant correlations between microstate features and scales within the stroke group. These results might provide some neurological evidence of EEG microstate analysis for stroke rehabilitation. Resting-state EEG microstate might assist clinical diagnosis and assessment application as a neurological marker.

## Data availability statement

The original contributions presented in this study are included in the article/supplementary material, further inquiries can be directed to the corresponding authors.

## Ethics statement

The studies involving human participants were reviewed and approved by the Ethics Committee of Tianjin University and Tianjin Hospital in Tianjin, China. The patients/participants provided their written informed consent to participate in this study.

## Author contributions

ZW, LC, and DM were involved in the conception and design of the study, data interpretation, and critically reviewed the manuscript. ZW, SL, MX, FH, and ZL participated in the experimental data collection of this manuscript. ZL and ZW were involved in the manuscript drafting and revision. ZW was involved in the data analysis for this manuscript. All authors contributed to the article and approved the submitted version.
